# Ecological interdependencies and resource competition: The role of information and communication in promoting effective collaboration in complex management situations

**DOI:** 10.1371/journal.pone.0225903

**Published:** 2019-12-17

**Authors:** Matthew Osborne, Emma Sundström, Örjan Bodin

**Affiliations:** 1 Stockholm Environment Institute (SEI), Behaviour and Choice Initiative, Stockholm, Sweden; 2 Stockholm Resilience Centre (SRC), Stockholm University, Stockholm, Sweden; Middlesex University, UNITED KINGDOM

## Abstract

Communication between resource users has repeatedly been shown to be of significant importance in environmental management. The proposed causal mechanisms are numerous, ranging from the ability of users to share information to their ability to negotiate solutions to common problems and dilemmas. However, what is less known is under what conditions these potential causal mechanisms are important and if, in cases when different means other than communication were available, whether they would be more effective in accomplishing these objectives. An example of such an alternative could be that instead of (or in addition to) users being reliant on within-group communication to acquire useful information an intermediary—such as a public agency—could provide that for them. Furthermore, the different causal mechanisms making communication beneficial might not be independent, neither in respect to each other, nor in respect to other externally imposed means to facilitate better environmental management, and not in regards to different contextual factors. This study makes use of laboratory experiments in an innovative way to explore these questions and specifically test the relative importance of communication in managing *complex* social-ecological system characterized by common-pool resource dilemmas, ecological interdependencies, and asymmetric resource access–all characteristics being present simultaneously. We find that when resources users are confronted with such a complex challenge, the ability to communicate significantly increases individual and group performance. What is more surprising is the negative effect on overall outcomes that providing external information has on outcomes, when the users also have the ability to communicate. By analysing the content of the conversations we are able to suggest several possible explanations on how the combination of external information provisioning and user communications act to increase individual cognitive load and drives intra-group competition, leading to a significant reduction of individual and group outcomes.

## Introduction

The critical role of the communication amongst resource users, stakeholders, and different kinds of beneficiaries and authorities in facilitating sustainable management of the environment is firmly established in research and practice [1,2]. The suggested causal mechanisms linking communication to improved management are plentiful, but less is known about when and under what circumstances one causal pathway is more important than another [3]. This presents not only a theoretical puzzle for scholars, but it also makes it more difficult to devise new policies and management approaches that are able to effectively address the many challenges that characterise environmental management/governance. In this study we will approach the question why exactly communication is important taking a stance in two broadly defined and widely used theoretical frameworks, ecosystem-based management (EBM) and common-pool resource (CPR) theory.

As a framework, EBM emphasises the importance of finding institutional, administrative and scientifically grounded ways to manage the *complete* ecosystem instead of concentrating on the management of often-arbitrary geographic units that may be the product of political or administrative requirements rather than social-ecological realities [4–6]. As such, the EBM framework overtly attempts to engage with the complexity and uncertainty inherent in the management of social-ecological systems; therefore the need to continuously learn and adapt is strongly emphasized [7].

CPR dilemmas are specific types of social dilemmas that explore the tensions between individual and collective incentives for maintaining a renewable resource stock in an environment where the ownership of the resource is shared or imprecisely defined [8]. Under such conditions, and particularly in the case of larger groups, early theoretic predictions of behaviour suggested that all individuals would seek to maximize their own resource extraction over equitable collective utilisation and the long-term sustainability of the resource [9,10]. This research concluded that private ownership would be the most effective, and possibly only way, to avoid resource overuse.

Over the last decades, research drawing on these two frameworks has shown that social-ecological systems can be sustainably managed even though formal ownership is not private [11–15]. A crucial finding taken from this body of empirical work was to elevate the importance of communication among users and stakeholders as an enabler of collective action [16]. Indeed, a key factor explaining observed users' ability to successfully manage CPR dilemmas in particular, was that they were able to collaborate and together devise common rules and procedures as to how the common resources should be managed [17]. This also involved developing and maintaining commonly approved practices for monitoring and enforcements. All of which would be impossible to achieve unless relevant actors have the capacity to communicate effectively. By ‘effectively’ we specifically focus on communication as a means to (a) develop, over time, trust and mutual respect among competing actors with sometimes conflicting goals and beliefs, (b) by drawing from such state of mutual trust, negotiate and jointly develop solutions to problems and dilemmas (cf. [18,19]), and (c) finally to facilitate improved monitoring and sanctioning based on the assumption that information about potential breaks with norms and rules can be both acquired and spread throughout a user community through communication. These findings have been investigated in detail by numerous experimental studies, which have identified the significance of a range of factors for encouraging the development of institutions regulating resource harvesting [17,20–27]. Despite this, which causal mechanisms are more important under which conditions remains under researched; indeed against general assumptions, some authors have demonstrated that, undirected, stakeholder collaboration can impair environmental management [3,15,28].

**Fig 1:**
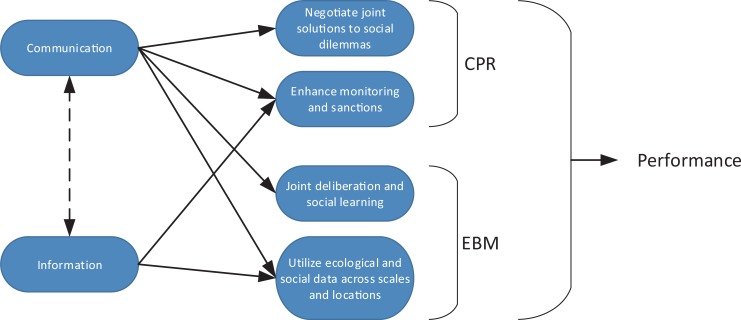
Conceptual description of presumed causal relationships linking communication, and information, to social-ecological outcomes. Communication is assumed to contribute to all four factors, all being assumed to contribute to better social-ecological outcomes (performance). Information, in isolation, is however only assumed to be related to two of these factors. The dashed arrow between communication and information symbolize the potential interaction effect between communication as a venue for disseminating information versus information as such (provided by a neutral and reliable third party).

We wish to address this gap by using an experimental approach to focus on how the ability to communicate can produce improvements in environmental management under the conditions of *ecological complexity and uncertainty* (described more in-depth below, and in the methods section). This condition provides for a context that not only resembles most if not all real-world management situations, but also where the different causal mechanisms addressed in the frameworks of EBM and CPR would both be expected to have a substantial role. In particular, we aim to disentangle the two different but presumably positive effects of communication in a complex environment that these frameworks tend to emphasise, i.e. the ability to facilitate joint (social) learning and the ability to jointly solve social dilemmas respectively, while also separating these effects from the more simplistic function of communication as a means to exchange information (see Fig 1).

### Dealing with complexity in environmental management research

Whilst there exists a vast experimental literature that points to the significance of communication and information as factors that improve individual and collective outcomes in a variety of games and settings [18,22,29–31], with notable exceptions, there are few studies that have looked at these questions with, as we argue here, a sufficient appreciation for the complexity and uncertainty implicit in contexts characterized by high levels of ecological complexity [32,33,27]. The orthodox requirement to maintain control to enable rigorous identification of causal mechanisms encourages experimental studies to reduce and simplify their set-ups to the minimum required to test the impact of changes to specific individual factors on observable outcomes [34]. Our objectives, however, involving investigating the different benefits of communication as emphasized in the different literatures of CPR and EBM makes such streamlined experimental design unfeasible. Instead, we argue our study complements previous studies in that we allow ourselves to move beyond the factor-by-factor design. As such, this study is sympathetic, to a point, with the arguments put forward by Schilnder [35] and Kinzig, et al. [36] who argue that experimental studies at less than ecosystem level maybe inappropriate due to their typical failure to accommodate the dynamic complexity of such settings.

Conversely, those studies that tend to be more comfortable with the challenges of researching human and ecological complexity–classically case-study based approaches–are faced with their own significant limitations. Case-study research requires a substantial investment in time and resources both in the planning and identification of suitable sites as well as in the data collection phase itself [37,38].

In an explicit attempt to find some middle ground between these different research approaches, we will deploy experiments in a less common way that enables us to test the effect of information and communication on a modelled social-ecological setting where we have deliberately aimed multiple factors to be simultaneously ‘in play’. Essentially we will, by design, create an action situation that more closely resemble a real-world environmental management scenario. Specifically, we use the interdependence of resources accessible to the different actors to create uncertainty and increase the complexity of the decision-making environment (see Methods below). Whilst such an action scenario provides greater control than a case-study study approach, its engagement with complexity and multiple concurrent factors poses difficulties in quantitatively tracing the exact cause and effect pathways–thus our objective becomes at least as much a structured approach to further hypothesis generation as it is to formally test effects.

## Methods

### Experimental design

Our experiments focused on the effect of information and communication on individual and group level resource-management. The game itself is similar in design to that presented by Lindhal et al,. [33] in that it creates an environment where participants are presented with a decision-making setting that is situated within a simplified but complex resource-harvesting environment. The setting requires the participants to be reliant on some level of experimentation to understand the nature of the task and one where individual outcomes are affected by the interaction of other users actions and the environment. The game was computerized, and the participants conducted the game using a graphical user interface on their individually designated computer in a laboratory environment. Prior to initiating the experiments, the study was reviewed by several colleagues that at the time of the study were establishing Stockholm Resilience Centre Ethics Committee. The committee was formed to, among other things, ensure compliance with The European Code of Conduct for Research Integrity. The outcome from these consultations was that the study would not gather any sensitive information. Therefore, and in accordance with ethics practices at Stockholm University and Swedish legislation, the study was exempt from formal ethical oversight.

**Fig 2:**
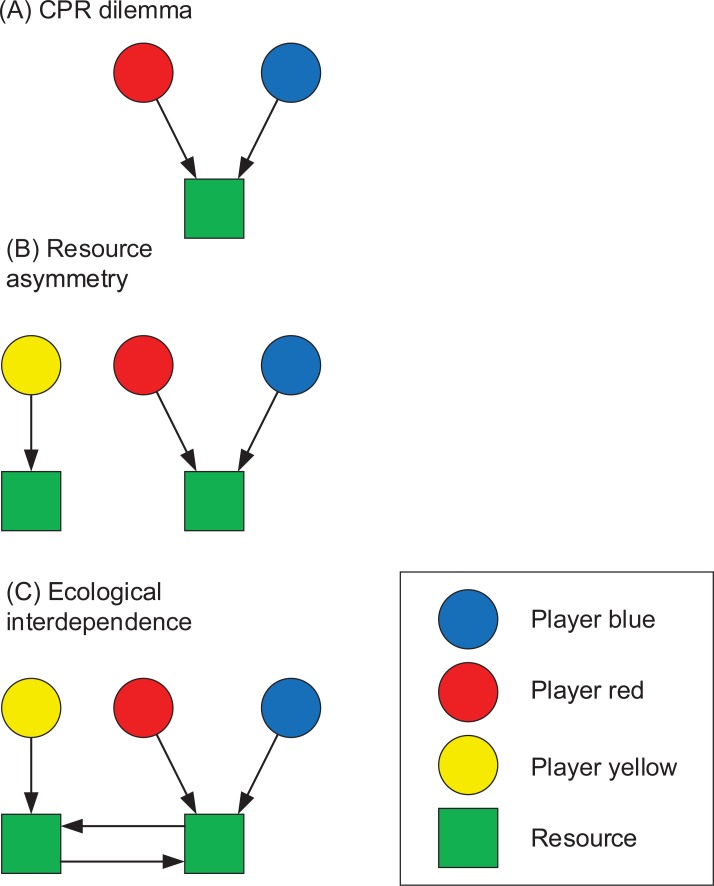
Graphical representation of the experimental design. A-C illustrates how common-pool resource dilemmas, asymmetries in resource access, and ecological interdependencies where added and combined to form our experimental base configuration (C). The different treatments where then applied on the base configuration by providing the players with information about others, and by giving them the ability to communicate.

Our experimental participants were 138 students enrolled at Stockholm University (see summary statistics in S1 Appendix). They were recruited using a web-based and pre-existing service where students voluntarily sign up as potential participants in ongoing and future behavioural experiments (at the time of the study approximately 5000 students were registered). Participants were organised into 46 groups of 3 participants per group (yellow, red and blue in Fig 2). All participants were recruited by sending out invitations to a random sample from the database. Upon arrival all participants were given a presentation that explained the game instructions and detailed their task. Informed consent was obtained verbally from all participants during experimental registration and briefing. The anonymity of the group member’s identities was ensured through the private selection of a game-card upon which was written an individual login username and password (each linked with a yellow, red or blue identity in Fig 2 –the experimental setup will be further described below). After the briefing, participants were randomly allocated to computer terminals and asked to log into the game platform. In this way there was no possibility that the participants could know with whom they were paired; and indeed, when communication was allowed the participants would universally refer to each other as the “red”, “blue”, or “yellow” player.

The exercise was separated into a number of rounds or ‘days’ in which participants were tasked with making a decision about how many hours they wished to spend harvesting from ‘their’ resource–to make the task less abstract we described each resource in Fig 2 as separate, but interconnected, forest-ecosystems. Participants could work a maximum of 20 hours each round and acquired harvests were recorded individually once all group members had made a decision. The relationship between harvest efforts and acquired harvests was non-linear essentially following parabolic form (“inverted U”, see Ecological Model in S1 Appendix). The key problem for the participant was hence to identify the peak where a certain effort gained maximum harvest-yield. This functional form resembles the idea of maximum sustainable yield where the ideal harvest level of a renewable stock should be aligned with the maximally possible reproduction rate; if too low the stock is under-utilized and if too high, the stock will eventually be depleted. The users sharing a resource (red and blue in Fig 2) would share the harvest, and the effort would be the sum of their individual efforts. They would share the harvest in proportion to their own harvest efforts, thus if one of them was spending more hours harvesting than the other, he/she would gain a larger share of the harvest. In this way we created a basic common-pool resource dilemma for red and blue players.

The harvests from the two resources were furthermore not independent. In short, the further away from the optimum (peak) one of the resources was harvested, the lower the maximally possible harvests that could be gained from the other resource. In this way we implemented ecological interdependency between the different resources: mimicking the characteristics of a basic ecosystem. The remuneration rates were thus dependent on a function of the resource utilisation of all users in the group.

Depending on the treatment, the participants would have the opportunity to see information about their own efforts and harvest outcomes as well as (conditional on the information treatment) the efforts and acquired harvests of the other players in their group. Conditional on the communication treatment, a chat-messaging box was present in the interface that allowed participants to talk within their group at any point they wished. Participants had the option to direct their messages to specific individuals or to all group members. Participants were told that the game would last a maximum of 30 rounds but were not told the precise number to avoid end-of-game behaviour—the actual number of rounds played was always 22. After completion of the last round the participants would automatically be forwarded to an online survey. Having completed the survey, each participant was called up and paid their earnings in private.

Some simplifications were necessary to reduce the manageability of the game for the participants. Firstly, though the game was framed in terms of hours spent harvesting resources; no cost was associated with the amount of effort invested by the players. Hence, the puzzle was ‘simply’ to find the peak of the inverted U functional form between efforts and harvests (it was not simple since the peak was partly a function on how well the other resource was managed). Secondly, participants were told that the ecosystem would return to its original state at the end of each round–meaning that the previous round’s harvesting efforts would have no impact in the capacity of the resource in the following ‘round’. These simplifications meant that the majority of groups were able to come to some understanding of these ecological dynamics through experimentation and/or social learning during the course of the game.

### Treatments and predictions

The study made use of four different experimental treatments. The first treatment (A) was the most basic whereby participants would not be able to communicate nor would they have access to information about the other participant’s performance. Our expectation was that without the access to information about others’ whereabouts participants would have to rely solely on individual experimental learning to understand the mechanics of the game; a task that is significantly complicated by the interdependencies between the resources as well as the sharing of one of the resources. Further, it would be virtually impossible for the players to coordinate their actions effectively, to establish trust, a group identity, or verify collective norms in the absence of communication (none of the factors in Fig 1 are covered for this treatment). Consequently, we anticipated that a typical group of conditional co-operators would struggle to gain understanding of the problem and in addition would struggle to interpret other players behaviour as cooperative resulting in the lowest overall outcomes [39,40]. The second treatment (B) continued to restrict communication between the participants but provided them with externally provided and objective information about the efforts and harvests of other group members. Hence, in this treatment individuals’ abilities to gain a better understanding of the ecological dynamics resulting from the interacting ecological components was thought to increase (two out of four factors in Fig 1 are covered). The third treatment (C) was a mirror of treatment B in that they participants could now communicate with one another but would not have externally provided information about their partner’s harvesting decisions and performance. Whilst we know that such information is of significant value, the users could however voluntarily communicate this information to the other participants. Further, communication’s ability to facilitate a collective mechanism that improves coordination, deliberation, negotiation and monitoring was likely more important than information alone (all factors in Fig 1 are covered)[20,22,30,31,33]. Given that, we anticipated that Treatment C would outperform Treatment B. The fourth and final treatment (D) was to allow full access to externally provided information and full communication between the participants. Our logical assumption was that groups within this treatment, where all factors of importance for EBM and CPR listed in Fig 1 are being covered at maximum strength, would outperform all other treatment groups.

### Experimental components

#### Complexity

Our experimental set-up includes three features typically found within real-world environmental management examples: common-pool resource dilemmas, asymmetries in resource access between users, and ecological interdependencies creating considerable uncertainty in regards to ecological dynamics.

As described in Fig 2, we first begin with the most simplified form of the classic CPR dilemma (a) whereby two users both have access to a single shared resource. In this way, harvesting efforts by one play will impact the outcomes for the paired player, and vice versa.

The second feature we include is to have asymmetrical access to resources by the different game users (b). Although differences in the degrees and modes of access to resources between multiple users are a common governance challenge in the real world, experimental studies often, but not always, assume symmetry of access [19,41,42]. To capture this asymmetry we increase the number of resources to two and the number of game players to three. As seen in Fig 2, the yellow player on the left has sole access to Resource 1 whereas the red and blue players share access to Resource 2 on the right in a classic CPR dilemma setting. Thus, we have implemented an in-built and non-negotiable difference in the ways the respondents can and will utilize resources.

The third component adding to the complexity of the set-up is that the two resources are ecologically connected (c). As often occurs in reality, the way in which users manage one resource will impact the ecological status and thus productivity of neighbouring and ecologically interconnected resources. Since the resource users will not know the strength and nature of this relationship, such interconnectedness not only increases the uncertainty of the ecosystem dynamics (i.e. two interconnected ecological resources resembles the idea of an *ecosystem*) and thus how to optimize harvesting efforts, it also increases the incentive for the users controlling the different resources to establish some form of collaboration between themselves—both as a means to gain knowledge about the system and to coordinate their management efforts.

Thus in summary we have an experimental set-up that captures the fundamental characteristics of a complex environmental management scenario in as simplified a manner as possible. On this ‘platform’ (base configuration) of complexity we will apply different treatments varyingly allowing for communication among the respondents, while simultaneously varying access to externally provided information.

#### Communication

We created the experimental structure in order to necessitate the participants to communicate and collaborate (to some extent) in order to resolve the challenges of the complex situation in which they find themselves. Communication was made possible through a chat-messaging interface. By recording the chat communication of the participants as they interact throughout the game rounds we are able to gain qualitative insights into how the social dynamic and structural properties of the action situation interact to hinder or facilitate collaboration. At the same time we make use of the quantitative experimental results to explore potential casual relationships between communication, information/knowledge and effective ecosystem management.

#### Information access

By controlling the amount of externally information that the participants have access to, we are able to control one key factor that communication is thought to contribute to. Essentially, if the sole benefit of communicating were to provide users with access to information (by sharing and exchanging information about each other’s harvesting efforts, acquired harvests etc.), the ability to communicate would be of no benefit if such information were externally provided ‘for free’.

### Data analaysis

#### Quantitative data analysis

We use two metrics for success in the experiments. The first is a simple *mean_coin_harvest* variable, which is the amount of gold coins earned by one individual in a given time frame, i.e. one round or game "day" (the acquired harvests were measured in terms of how much earnings they gave to the extractors). The second metric is *optimal_difference*, which is the amount of hours away from the optimal harvesting rate per round for each player. Optimal harvesting rates were 7 hours each for the red and blue players (14 together) and 11 hours for the yellow player. This means that increases in *mean_coin_harvest* and decreases in *optimal_difference* would indicate better outcomes. We chose these two performance measures since they complement each other. Optimal difference is better than *mean_coin_harvest* to statistically capture small performance differences when the respondents are close to the optimal harvests peak, where the *mean_coin_harvest* variable numerically do not vary so much (the peak, following the shape of a gauss curve, is rather flat). Optimal difference can thus qualitatively be seen as a “linearization” of the *mean_coin_harvest* variable that, close to the peak, follows a parabolic shape. Furthermore, the *optimal difference* variable, as we constructed it, assume that the common-pool resource should be split equally between the red and the blue player. Hence, by using this performance indication, we also were able to capture an assumption of what constitutes a preferred strategy where both these players would benefit equally much from the shared resource.

Both *mean_coin_harvest* and *optimal_difference* were summarized per group, and per round of the game. These group means, on a per round basis, constituted the dependent variables capturing performance in further analyses.

We used regression analysis to test whether if the different treatments (A-D) affected performance. To control for the groups’ abilities to improve their performance over time, round was included as a control variable (co-variate). The expected time dependency made a simpler t-test unfeasible. We performed these regressions in a pair-wise fashion where we tested one treatment versus another, in total 6 comparisons (A vs. B, A vs. C, A vs. D, B vs. C, B vs. D, C vs. D). Since we applied one regression analysis per pair (in total 6 pairs), p-values need to be adjusted accordingly (with many tries, the likelihood for a p-value to get below 0.05 is higher than one in twenty). The most conservative approach it is reduce the threshold for statistical significance by dividing 0.05 with the number of models (6). We also used the Benjamini–Hochberg method to take into account multiple models, while explicitly allowing for a false discovery rate of 25%.

We used explorative regression analysis to test if the occurrence of different discussion topics (described below) were statistically related to our two outcome variables, *mean_coin_harvest* and *optimal_difference*. We tested these relations while taking potential differences between groups as well as the time periods themselves into account (using the random effect panel data regression model in the R-package plm [43])). In that way we were able to control for group heterogeneities while also exploring if there are relationships that are only specific to certain phases of the experiments. We also controlled for the total number of messages per round for each group (one instance of a topic code, see below, is representing one message). P-values were treated following the Benjamini–Hochberg method. All statistical analyses were conducted using the package plm [44,43].

#### Qualitative data analysis

As a part of our attempts to disentangle causal mechanisms, in addition to the quantitative analyses we also qualitatively analysed the content of the communication among the participants (treatment C and D). In this way we are able to better grasp what kind of processes were at play, and hence seek explanations in terms of why they were behaving in certain ways.

To explore the actual content of the conversations we use an adapted analytical approach to that was first presented by Pavitt [45] and used in other resource-experimental studies [46,47]. This approach requires that after completion of the experiments, the transcripts of the group-chat conversations were downloaded and each independent message sent by a participant was separated into independent individual message units. Two members of the research team independently coded all chat messages in a group’s conversation against a coding framework of six different subject areas–Topical, Functional (Substantive), Functional (Maintenance), Social-Ecological Links, CPR Group & Harvesting Strategy and their associated sub-categories (see Table 1). In practise this meant assigning a code-value to each message unit up to six times–for each of the six different subject areas. Often the content of a particular message would have no relevance to a particular subject, in which case the message unit would have no value assigned to it. Once completed, a random sample of approximately 10% of each group’s conversation’s coding was compared between the two coders to verify reliability. Overall coding reliability was extremely consistent with matching found to be greater than 90% throughout.

**Table 1:** Coding, CPR and EBM factors, and descriptions.

Subject area	Sub-category	CPR/EBM factors^1^	Unit Description
Topical	Game Understanding	Joint deliberation	Discussion relevant to the rules of the game, with the general intent of increasing game players’ understanding of how the game is played. Also, nature of game-setting/scenario and rewards.
	Past Round	Utilize social and ecological data, Enhance monitoring and sanctioning	Discussion relevant to what occurred during past rounds in the game. Emphasis on game outcomes in pervious rounds.
	General Strategy	Joint deliberation, Negotiate joint solutions	Discussion relevant to the general strategy to be used in subsequent rounds. They do not include discussion relevant to specific proposed strategies, i.e. when specific harvesting figures are mentioned.
	Specific Strategy	Joint deliberation, Utilize social and ecological data	Discussion relevant to specific proposed strategies; that is, proposals including specific numbers of points to be harvested.
	Off-topic Tangents	N/A	Discussion of non-game relevant subjects
Functional: Substantive	Information	Joint deliberation	Statements about the nature of the game situation that are essentially objective and descriptive, along with acknowledgments following those statements.
	Suggestion	Negotiate joint solutions	Statements that introduce or ask for a proposal, along with acknowledgments following those statements.
	Computation	Joint deliberation, Utilize social and ecological data	Statements that ask for or are part of calculations relevant to proposals, along with acknowledgments following those statements.
	Elaboration	Joint deliberation	Nonevaluative statements about previously offered proposals and their consequences. General/Non-specific
Functional: Maintenance	Positive	Joint deliberation, Negotiate joint solutions	Statements showing pleasure, joking, or positive response to expression of pleasure and jokes. Positive maintenance units can also indicate affiliation or social support for other group members, or identification with or praise for group as a whole. Finally, positive maintenance units may consist of positive responses to episodes of tension or antagonism.
	Negative	Joint deliberation (-), Negotiate joint solutions (-)	Statements of disapproval or criticism of the group or other players, or expressions of nonconformity with the other players, along with direct responses to these statements. Negative maintenance units can also show displeasure, frustration or disinterest, and acknowledgments of incompetence.
	Procedural/Group Learning	Joint deliberation, Negotiate joint solutions	Assigned if the messages are concerned with the process by which the decision is made—the status of the group and identification with the group. This would include attempts to guide the discussion. Emphasis on the development of group identity: the stressing of 'we' over 'I' and group-commitment. Evidence of learning behaviour from past experience infuencing future decisions.
	Sub-group Formation	Negotiate joint solutions (-)	The development of a 'them' and 'us' situation.
Social Ecological Links	Awareness	Joint deliberation, Utilize social and ecological data	General awareness/comment of the social-ecological link (awareness) Neutral or Positive. Understanding
	Misunderstanding	Joint deliberation (-)	Confusion of the social-ecological link (complexity)
CPR Group	CPR group identity	Negotiate joint solutions (-)	CPR Group self-identification (or vice versa): Red and Blue vs. Yellow
	Tension caused by CPR (Red/Blue)	Negotiate joint solutions (-)	Tension/Competition between CPR group (as opposed to general tension between all players)
Harvesting Strategy	Innovation	Joint deliberation, Utilize social and ecological data	Not general but new specific strategy proposed.
	Evolution	Joint deliberation, Utilize social and ecological data	Suggested adaption of existing strategy.
	Revolution	Joint deliberation, Utilize social and ecological data	Rejection of old strategy and proposal of entirely radical new strategy

^1^These factors relates to the CPR and EBM factors in Fig 1 (column 2). If the relationship between the sub-category and the relevant CPR/EBM factor is inherently negative, meaning that chat exchanges coded as this sub-category are having a negative effect on the factor, a minus sign is added. Please observe that the sub-categories are not necessarily exclusively linked to the outlined CPR/EBM factors, we have only emphasized what we deem being the strongest links.

The choice of Topical and Functional subjects was made using insights drawn from the substantial body of theoretical work that explores what important processes that influence individual decision-making within a collective-action setting (see [8,48–51]). In particular, we were interested in finding evidence for the processes of social and experimental learning within a resource dilemma setting [52–54], the establishment of interpersonal trust between participants [21,55–57] and the development of a collective group identity [58,59]. After coding of chat messages on these well-documented processes, we inductively selected the additional three categories for examination–Social-Ecological Links, CPR Group & Harvesting Strategy. The identification of these final three subjects was prompted by our initial coarse grain analysis of all communication messages, and our specific interest in these aspects of the decision-making environment. Social-Ecological Links refers to comments directly attributable to the structural linkages between the different the resources and the players. CPR Group refers to comments that directly identify the significance of the relative degree of interdependence between the blue and red players relationship in regards to the (relatively) independent agency of the yellow player. Finally, the Harvesting Strategy refers to comments that explicitly regarded the players' reflections on their resource extraction strategy. Further explanation of each of the sub-categories is provided in Table 1. Finally, we linked each sub-category to the different factors of CPR and EBM emphasized in Fig 1. In that way, we were able to interpret the results from the qualitative analyses in light of both the EBM and CPR frameworks.

Following this coding procedure, we could assign a numerical value to each group capturing how many times they exchanged messages during the duration of the game corresponding to any of the sub-categories. To investigate if there are any statistical significant differences between the treatments, we then carried out Welch’s t-test for each message topic.

## Results

### Quantitative results for the different treatments

The descriptive results broadly confirmed our expectations (providing external information and communication abilities generally improved performance)(Table 2). However, there was no notable improvement by just adding external information (B) for *optimal difference*, but *mean_coin_harvest* improved in comparison to the no information and no communication treatment (A).

**Table 2:** Treatment information, group numbers and performance.

Treatment	Info	Comm	*mean_coin_harvest*					*optimal_difference*				
			Obs.	Mean	Std. Dev	Min	Max	Obs.	Mean	Std. Dev	Min	Max
A	No	No	154	11.90	7.42	0	24.7	154	3.57	1.73	0.67	10.67
B	Yes	No	264	14.30	7.07	0	25	264	3.56	1.72	0	8
C	No	Yes	176	19.11	5.87	0	25	176	1.94	1.10	0	8
D	Yes	Yes	418	17.47	6.92	0	25	418	2.72	1.81	0	10

Table 3 presents the results from the tests of how the performance effects of the treatments differed between the different treatment pairs. As expected the least well performing groups are those from treatment A—which was the baseline treatment for the experiments. Given the complexity of the challenge and the inability for participants to communicate and coordinate their efforts it is no surprise to see these groups do least well. Treatment B groups improved their overall *mean_coin_harvest* which suggests that they were able to make use of the external information to better understand the decision environment (the effect on *optimal difference* was however not significant). What is surprising is that the groups that did the best were not those that had access to external information and were allowed to communicate (D) but rather the most successful where those groups that were allowed to communicate but were not provided with information about the earnings and efforts of other group members (C).

**Table 3:** Pair-wise tests of the treatments effect on performance.

Pair	Coefficient	Pair	Coefficient
	*mean_coin_harvest*		*optimal difference*
A versus B	-2.40*	A versus B	0.015
A versus C	-7.21**	A versus C	1.63**
A versus D	-5.56**	A versus D	0.85*
B versus C	-4.81**	B versus C	1.61**
B versus D	-3.17*	B versus D	0.83*
C versus D	1.64*	C versus D	-0.78**

*Significant assuming a threshold of 0.05, while taking into account multiple models and allowing for a false discovery rate of 25%

**P-value less than 0.05/6 = 0.0083.

Generalized least squares (GLS) for panel data with Huber-White robust standard errors were used using the software program STATA.

### Communication analysis (treatment C and D)

The results from the regression analysis of the relationships between the content of group discussions and their performance are presented in Tables 4 and 5 (using the package plm in R, see methods). The tables also include information on how many chat message of each topic were sent for any given game (i.e. 22 rounds with a group of three individuals). We also included the time (rounds) in the regression models (as 21 dummy variables, with round 1 as the base). We did so since for both treatments; there was a strong and significant effect of time on performance (the effect of time on performance was also revealed in the pair-wise regression analyses, although not shown). This was as expected as participants learn over time. However, it was clearly observable how strongly time explained performance differed between the treatments. We present the results from the regression analysis only taking time and total number of messages into consideration in S1 Appendix. Without information (C), a significant effect of time on performance is seen as early as the third round, and then the effect steadily increased and reached a coefficient value of approximately 2.2 (Regression Results in S1 Appendix). In contrast, with information (treatment D) the significant effect showed up first at round seven (Regression Results S1 Appendix). And the coefficients were typically in the order of 0.8 to 1.4, essentially approximately 50% in comparison to treatment C. In addition, the adjusted R^2^ for treatment C was 0.27, whereas it was only 0.14 for treatment D. Hence, these results indicate that if respondents are not given externally provided information, they performed steadily better over time, and that time itself is a fairly good predictor of performance. With information, the time-based performance increase is less steady, not as strong, and the model explains less of the variability in performance.

**Table 4:** Treatment C, the performance effect of the number of topical chat messages for any given round.

	Mean_coin_harvest			Optimal_difference			Mean messages / game
Chat topic	Coeff	p-value	adj p-value^1^	Coeff	p-value	adj p-value^1^	
**game_understanding**	-0,18	0,79	0,2	0,103	0,412	0,075	5
**past_round**	-0,193	0,528	0,1	0,011	0,84	0,188	44,75
**strategy_general**	**0,486**	0,19	0,05	**-0,107**	0,121	0,013	16,75
**strategy_specific**	-0,069	0,823	0,213	0,019	0,745	0,15	51,5
**off_topic**	0,001	0,998	0,25	0,015	0,9	0,213	5,25
**information**	-0,218	0,752	0,175	0,085	0,507	0,113	4,5
**suggestion**	0,227	0,439	0,088	-0,037	0,502	0,1	65,88
**elaboration**	-0,226	0,539	0,113	-0,003	0,965	0,25	18,38
**computation**	-0,056	0,86	0,238	0,017	0,769	0,163	27,63
**positive**	0,543	0,262	0,075	-0,064	0,481	0,088	11,25
**negative**	**-2,238**	0,094	0,038	**0,348**	0,163	0,025	1,75
**group_learning**	0,148	0,741	0,163	-0,039	0,641	0,125	27,63
**subgroup_formation**	-0,624	0,66	0,138	0,233	0,376	0,063	1,13
**awareness**	0,76	0,676	0,15	-0,042	0,902	0,225	1,13
misunderstanding*	1,226	0,757	0,188	-0,048	0,948	0,238	0,25
**cpr_identity**	**-2,029**	0,088	0,025	**0,309**	0,163	0,038	3,25
cpr_tension*	**10,558**	0,055	0,013	0,43	0,675	0,138	0,13
innovation*	0,927	0,612	0,125	-0,087	0,799	0,175	0,88
**evolution**	-0,977	0,234	0,063	**0,17**	0,267	0,05	9,25
revolution*	0,373	0,85	0,225	0,053	0,886	0,2	0,75
**R2 (mean for all models)**	0,27			0,27			

^1^Adjusted p-value based on a false discovery rate of 0.25, using the Benjamini and Hochberg [1995]

*Very few datapoints, caution warranted

**Table 5:** Treatment D, the performance effect of the number of topical chat messages for any given round.

No information	Mean_coin_harvest			Optimal_difference			Mean messages / game
Chat topic	Coeff	p-value	adj p-value^1^	Coeff	p-value	adj p-value^1^	
**game_understanding**	**-0,774**	0,032	0,038	**0,326**	0	0,013	6,95
**past_round**	0,04	0,776	0,188	-0,001	0,973	0,25	57,16
**strategy_general**	-0,268	0,21	0,075	0,093	*0*,*091*	0,088	26,32
**strategy_specific**	0,145	0,379	0,138	-0,068	0,11	0,1	61,89
**off_topic**	-0,098	0,819	0,2	-0,032	0,771	0,213	4,42
**information**	**-0,9**	0,008	0,013	**0,3**	0,001	0,025	6,37
**suggestion**	-0,011	0,942	0,238	-0,004	0,911	0,238	86,05
**elaboration**	0,206	0,353	0,125	-0,063	0,274	0,138	21,16
**computation**	-0,022	0,893	0,225	0,017	0,684	0,2	33,16
**positive**	0,194	0,418	0,15	-0,089	0,154	0,113	16,89
**negative**	-0,555	0,252	0,1	0,092	0,464	0,15	4,47
**group_learning**	**0,572**	0,013	0,025	**-0,15**	0,011	0,038	35,21
**subgroup_formation**	**-1,513**	0,039	0,05	0,337	*0*,*075*	0,063	1,26
**awareness**	0,168	0,836	0,213	-0,051	0,809	0,225	1,63
misunderstanding*	-0,694	0,672	0,175	0,526	0,215	0,125	0,58
**cpr_identity**	0,002	0,997	0,25	0,061	0,683	0,188	3,95
cpr_tension*	-3,137	0,188	0,063	**1,487**	0,016	0,05	0,32
innovation*	-1,26	0,307	0,113	0,206	0,518	0,163	1,11
**evolution**	0,504	0,244	0,088	-0,194	*0*,*084*	0,075	10,42
revolution*	-0,429	0,642	0,163	0,138	0,566	0,175	1,63
**R2 (mean for all models)**	0,12			0,15			

^1^Adjusted p-value based on a false discovery rate of 0.25, using the Benjamini and Hochberg [1995]

*Very few datapoints, caution warranted

We also carried out Welch’s t-test for each message topic, for the two treatment groups consisting of all games with communication, with and without external information, respectively. When analysing if the quantity of any of the message types differed between the treatments, messages of sub-category *strategy_general* where less common for treatment C (p-value 0.025). For sub-category *negative*, there was a marginally significant difference (p-value 0.075, with more negative messages in treatment D). Hence, there were no major differences between the treatments in terms of how often these different topics were communicated. However, for the sub-categories *cpr_tension* and *misunderstanding*, the total numbers of messages were low for both treatments. Thus, regression results for these sub-categories are not fully reliable. Since the number of games was lower for treatment C, the number of messages related to the topics innovation and revolution also became low, thus the result in Table 4 regarding these topics should be interpreted with caution.

There are some notable differences between the treatments. In addition to the different effects of time, for treatment C none of the topics had a significant effect on performance, whereas for treatment D five topics had significant effects (three for both performance measures, and two for either mean_coin_harvest or optimal difference). We argue this qualitatively difference between the different treatments being a finding by itself, which we elaborate further in the discussion. However, when applying a less strict criterion for statistical significance, five factors were shown to effect performance for treatment C (three for both performance measures, and two for either mean_coin_harvest or optimal_difference).

To provide a more qualitative understanding of the unexpected difference in performance between treatment C and D, we present some extracts from the discussions among the participants. We deliberately have selected examples that capture some potential explanatory mechanisms we elaborate further in the discussion. Table 6 presents extracts from the communication within a group given no access to externally provided information (treatment C). It illustrates a process where they collaborate by sharing information, agreeing on common strategies, and deliberate over the acquired results. Table 7 illustrates a group (treatment D) that struggles with some users consistently providing the others with false information, likely with the intention to get ahead of the others acquiring better harvests and/or acquiring a larger share of the common resource (the latter is supported since the persons providing false information are the red and blue players–the ones that are sharing a common resource). Finally, Table 8 presents the communication of a group (treatment D) that clearly had difficulties in making sense of the game. In particular, after round 9, it seems that the externally provided information creates more confusion than clarity.

**Table 6:** Chat 1—No Information, constructive discussion.

Round	Player	Chat Message
1	Yellow	hello, lets try to adjust the level of working hours so everyone get a good harvest
1	Red	Sounds good
2	Blue	Fine
2	Red	I had about 4 gold coins for 6h of work last time
2	Yellow	I think that any or both of you must have worked to much day 1
2	Yellow	i just got 1 gc
3	Blue	So we keep ourselves low at first
3	Red	I got none: (
3	Red	Worked 1 hour day 2
3	Blue	Worked 2 hours
3	Blue	this day
3	Red	worked 6 hours day2
4	Blue	Let's try 3 hours each this day
4	Red	Ok
4	Yellow	Ok, I will try with 3 hours for day 4
5	Yellow	Did you both have 3 hours day 4?
5	Red	I did
5	Red	yes
5	Blue	Three gold coins for me and red, 2 for yellow I had 3 hours for day 4. "
5	Blue	I suggest 4 hours for me and red and 5 for yellow
6	Red	ok, sounds good
6	Red	4 hours day 6
6	Yellow	I take 5h for this day then
6	Blue	This time I suggest 5 hours for me and red and higher for yellow
6	Yellow	think it is a good level if the harvest is over the number of worked hours
6	Blue	Let's see what happens
6	Red	:)
7	Blue	Success, we are raising, let's move one step up each day

**Table 7:** Chat 2—Information increasing competition and conflict.

Round	Player	Chat Message
14	Blue	hehe, i took 10
14	Yellow	So, blue liead and picked nine, red and I told the truth and picked 8.
14	Yellow	Okay then, ten. Liar!
14	Blue	nah, red picked 9
14	Red	we are good colors.
14	Blue	feeling blue
14	Yellow	All pick nine this time?
14	Red	oh right. but i wrote that and it was the truth!
14	Blue	okay, i will pick nine this time
15	Blue	why did you do differently without telling?
15	Blue	we cant measure results that way
15	Yellow	red's done differently every time
16	Red	i have been doing 8–11
16	Yellow	yes, but always more than you've been telling in advance–we can see!@!
16	Red	hehe

**Table 8:** Chat 3—Information increases cognitive load (especially after round 9).

Round	Player	Chat Message
5	Red	Well we all seem to do best day one, so maybe we should go with the same?
5	Yellow	total 29h day 1 —> 9h-10h each..
5	Red	Yes, so i took 10 this round
5	Yellow	. . .so if all try 9h ?
5	Red	i'll take 9 this next time
6	Yellow	i dont think its scales linearly thou
6	Yellow	it has a peak somewhere higher
7	Blue	hmm well, i have no idea on which combination of hours
8	Blue	if we write each day how many hours we take its easier to figur out
8	Red	the pay off for the same amount of hours must be difference since you two share a wood and i dont
9	Red	So "Red" what if we take the same amount of h ?
9	Yellow	im not sure if it looks right for you but on my screen my bars are one day wrongly placed to the right
9	Blue	not for me, your bar looks the same
10	Red	My bars is ok i guess
10	Yellow	yes, and after that I think by bars is one day delayed
10	Red	how come you can peak so high and i dont:(
11	Red	. . . .dont know..:-)
11	Blue	from the looks of it yours mine are more stable and youve have gotten a huge spike
11	Blue	soo if one of you pick a very low number the other one gets a lot of reward

## Discussion

Our results clearly demonstrate that communication provides more benefits than just functioning as a means of information exchange. The results in Table 2 shows that if groups are provided with external information, but with no ability to communicate (treatment B), they do not perform as well as the groups that are provided with the means to communicate but no external information (treatment C). Hence, such groups are able to utilize communication to achieve better performance that goes over and above the ability to share information. All of which is in line with our expectations given that communication is important both for enhanced (social) learning and achieving a better understanding of the resource dynamics (EBM) and for enabling users to collectively address common-pool resource dilemmas and challenges (CPR) (Fig 1).

We also demonstrate that providing information, in general, enhanced performance (Tables 1 & 2). But, we also show that if external information is provided together with the means for communication, performance actually decreases in comparison with treatments where only the means for communication was provided. Why we get these results, essentially demonstrating a negative interaction effect of external information and communication, is something of a conundrum. In similar studies, such as that by Janssen et al [25], the authors found that information and communication had a positive casual effect on outcomes–arguing that it allowed enforcement mechanisms to be effective thus allowing the conditional co-operators to drive positive outcomes for the individuals and groups. Our results agree with these findings up to a point, but the negative interaction effect suggests that these two factors do not monotonically increase performance independent of each other. Here we use our results from the explorative regression analyses and the qualitative analysis of the chat messages to better understand why this might be the case.

There are a number of potential explanations for this unexpected outcome, and as discussed earlier, our experimental design does not allow us to unambiguously identify the precise causal mechanisms. However, our results provide us with several clues that we here use to reconstruct possible explanatory mechanisms. In that sense, we use our results to construct hypotheses rather than to validate pre-specified hypotheses.

### The cognitive load hypothesis

We see a larger variability of performance increasing over time when communicating groups get access to external information. Since the only way to gain a better understanding of resource dynamics in our experiment is through experiential learning, we propose widespread confusion among the participants disturbs the learning process; thereby reducing performance. The confusion stems from the sheer amount of information, both produced through the exchanging of chat messages and provided externally to the point where it is generating detrimental cognitive loads. In effect we argue that we are observing the effect of increased cognitive load crowding out the benefits of communication and information to increased understanding (see [60,61]). In such a state of confusion, a marginally increased amount of information could further disturb the learning process. This could explain why we see an unexpected negative performance effects of an increased exchange of messages of the sub-categories *game understanding* and *information* (both related to the EBM factor “joint deliberation”, Fig 1) for treatment D, but not for treatment C (Tables 4 and 5). The communication extract presented earlier (Table 8) gives further support and indicates a state of confusion amongst the participants who have not been able to figure out how to best manage the fictive resources through the first eight rounds, and then at round nine when they actively start to discuss the information provided to them by the game design (in addition to the information they already have shared in their messages), that information seemingly adds to the confusion. The extract from a treatment C group (Table 6) presents an entirely different story in that the participants not only exchange information, buy they use that shared information to coordinate their individual activities and, over time, develop some joint understanding on how to perform better. If our suggestion is valid, this more constructive use of communication could then partly be attributed to a sparser amount of information that would reduce cognitive overload whilst facilitating the gradual accumulation of knowledge through experimental learning: all of which would very much align with the principles of continuous learning through experimentation found within the EBM framework.

At this point we need to clearly state that these two examples can perhaps be seen as two ends of a continuum, and there are several examples of groups with access to both information and communication that do better than the example in Table 8 (and vice versa). Nonetheless, we suggest that groups from treatment D tends to be closer to that end of the spectra, although we should be cautious about reading too much into this finding since this may well change if games were played longer (thus giving the participants more time to turn confusion into knowledge). An inverted u-shaped relationship between performance and the amount of received information might even be expected. Initially, as external information is provided in an environment where no information was available, the performance would increase (cf. treatment B versus A). However, that effect could at some point reach a peak and subsequent increases of information would actually decrease performance, as participants are less able to make productive use of the increased amount of information (treatment D versus C). The information received from others through communication, and from the game itself, would in combination put the participants on the far right side of the peak where poor performance is associated with information overload.

Furthermore, in this context it should also be pointed out that when groups with the communication and information treatment exchange messages related to group learning, they perform better (Table 5). However, that sub-category of messages (covering joint deliberation and negotiation of joint solutions) implies that the group is communicating to learn and deliberate about the group itself and how it should be organized

### The competition triggering hypothesis

So far, we have discussed the (individual and/or social) learning aspect of our experimental setup that is strongly articulated in the framework of EBM. Negative performance effects related to difficulties dealing with CPR dilemmas were, however, also observed. For treatment C, discussions about group identity (“us and them”) reduced performance, and for treatment D, discussions related to tensions between the users sharing a resource (red and blue, Fig 2) and the other having his/her own resource (yellow, Fig 2) increased the deviation from optimal effort levels (Tables 4 and 5). These results, for both treatment C and D, are in line with our expectations, except for the positive effect of CPR tension in treatment C, but this estimate should be treated with great caution due to the virtual absence of data(Table 4).

That does not however rule out that the CPR dilemma still has an role in explaining why treatment C gave better performance than treatment D. The blue and red players are sharing a resource, and the game’s reward mechanism stimulates the participants to get ahead of the others. Hence, there are incentives for participants to share false information about their own activities (and outcomes). This is clearly shown in our communication extract (Table 7). Further, since the participants lack any direct means of punishing liars (beyond attempts as social punishment, we see evidence of in Table 7), the ‘cost’ of being caught lying is limited (this lack has been shown to contribute to reduce performance in different CPR settings, see e.g. [32,62]. Furthermore, another associated cost with lying is that it is likely to reduce everyone’s ability to learn, resulting in a likely reduction in everyone’s earnings.

However, it this holds true, why would participants in treatment C not lie as well? Actually, since information is not provided externally in treatment C, it would be harder for them to reveal when others lie; thus decreasing participants’ incentives not to lie (albeit the limited abilities to sanction implies that the cost of being caught is, as stated above, not severely high). Although this suggests that participants in treatment C would be at least slightly more inclined to lie, our results nonetheless indicate that it is unlikely that they were lying that much since they were able to steadily improve performance over time. We instead suggest that in our experiment, when users are given externally provided information about others outcomes; it triggers competitive behaviours between the participants and invites them to provide incorrect information about your own earnings and efforts.

Our explanation for this goes as follows. If no external information is provided, the participants are less stimulated to act competitively, and instead are more inclined to reduce uncertainties through sharing of (correct) information and engage in joint deliberation and learning. This suggestion aligns with a hypothesis put forward in an earlier study stating that the need to gain understanding of complex resource dynamics overshadows the incentive to get ahead of others [33].The explanatory mechanism behind such a relationship could be that uncertainties about the resource dynamics makes it difficult for participants to predict the outcomes of their own efforts to get ahead of others. In other words, it will be hard to predict if you gain or lose by not engaging in joint negotiation and instead try to get ahead of others. This reasoning bears similarities with the broad idea that people that are faced with inherent uncertainties about their own outcomes in relation to others tend to choose strategies that would result in limited inequalities [63].

And as discussed earlier, if users are less inclined to share false information, learning as a group effort is enhanced since users do not have to spend cognitive resources on trying to make sense of incorrect information. The more you and the group learn about the game and the resource dynamics, the better you and the group are able to perform (by settling in on optimal effort levels and thus gaining maximum harvest yields). This could create an upward spiral of positive reinforcement of communication, learning,performance, and the collaborative social norms (cf. [64]). This is supported by our results in that the average number of messages carrying disapproval or criticism of the group or other players was, albeit only marginally statistical significant, higher for treatment D than for treatment C (Tables 4 and 5). Hence, when the participants eventually reach the point where uncertainties about the complex dynamics are no longer overshadowing the incentives to get ahead of other, they have in parallel developed strong group norms and mutual trust that make them less inclined trying to get ahead of others

## Conclusion

Our results show that both external information and communication, separately, enhance actors’ abilities to better manage complex resources characterised by asymmetries, ecological interdependences, and where an actor’s extraction of a resource has reduced the available amount that others can use. This confirms earlier research on the importance of information and communication for both improved EBM and to better deal with CPR dilemmas. Our results, however, also demonstrate a negative interaction effect between communication and externally provided information. This is a puzzling result, but by combining insights drawn from our explorative regression models that relate topics of conversations with performance, and a qualitative analysis of the groups’ discussions over time, we here propose some causal mechanisms that could explain these unexpected results (summarized as hypotheses 1–3 below):

When resource users are confronted with the challenge to manage complex ecological resources, giving them the ability to communicate while simultaneously providing them with externally derived information about each other’s performances will (1) increase their cognitive load making them confused and causes a reduction in their joint ability to learn effectively, and (2) triggers competitive behaviour that inclines them to use the communication ability to get ahead of others. If, however, external information is not provided, (3) the users will be less inclined to use the communication ability to get ahead of others, and instead they will be more inclined to engage in joint learning. That engagement will cause a positive and robust reinforcement of information sharing, learning, and development of mutual trust that will increase performance.

By linking these pending insights back to the overarching objective of our study, i.e. to better understand why and under which conditions communication would be of key benefit to either/both enhance EBM through social learning and/or to enable users to better deal with CPR dilemmas, we suggest the following. Under the condition with limited information (i.e. a state characterized by significant uncertainties about complex resource dynamics), communication both enhance social learning and thus EBM, and also helps users to better deal with CPR dilemmas by negotiating joint solution. The reason for this would at least partly be attributed to uncertainty itself, which makes users inclined to learn and build knowledge as a group, and to engage in jointly coordinated experimentation. Further, the competitive component of a CPR dilemma (i.e. a chance to get ahead of others, cf. Hardin [10]) is seemingly perceived as less important as joint negotiations seeking to establish a state where everyone is doing reasonably good, cf. Ostrom 1990). However, when external information is provided, the competitive component of the CPR dilemma seems to be given more weight in relations to the willingness to seek negotiated solutions. Thus, the contribution of communication to deal with CPR dilemma without extensive overharvesting is reduced. Further, although being given external information would not constitute any obvious reason for the users not trying to improve their understanding of resource dynamics, the social learning process might still be disturbed. The disturbances arise from two sources–the cognitive overload itself reducing abilities to deliberate, and through side effects of the competitive component of the CPR dilemma that is increasingly overshadowing the willingness to negotiate. The latter would essentially consist of the following: increased incentive for users to withhold or deliberately provide incorrect information (therefore also making actors more cautious about trusting what others are reporting), as well as making users less inclined to learn as a group and instead focus more on individual learning (as a way to get ahead of others). In conclusion, these pending insights would imply that the observed positive effects of communication for EBM and CPR are indeed in agreement with previous research, but they also suggest that these effects on EBM and CPR are not independent of each other, rather they interact. Especially under a condition where the competitive component of a CPR dilemma is driving user behaviours more than their willingness to engage in developing joint solutions.

We conclude arguing that our experiments reveal new insights into understanding the role of information and communication in social-ecological settings that incorporates a significant degree of complexity. The study also provides an illustration of an alternative approach to controlled one-factor-at-a-time experiments that continues to gain interest among scholars [27,33,46]. We finally suggest that the multi-factor approach is particularly useful when investigating the intriguing question as to whether there is an irreducible level of complexity, for different types of social-ecological systems settings, which if crossed essentially make a hard-driven reductionist approach more or less irrelevant.

## Supporting information

S1 Appendix Summary statistics, ecological model and supplementary results. (DOCX)
