# Supplementary material for: Ecological interdependencies and resource competition: The role of information and communication in promoting effective collaboration in complex management situations
Source: PLoS One. 2019 Dec 17;14(12):e0225903. doi: 10.1371/journal.pone.0225903 (PMC6917270; doi:10.1371/journal.pone.0225903)
Supplement: S1 Appendix — (DOCX) [file pone.0225903.s001.docx]

**S1 Appendix**

**Summary statistics**

| **Table A. Population summary statistics** | | |
| --- | --- | --- |
|  |  |  |
| **Variable** | **Mean** | **STD** |
| Age | 28.8 | 9.48 |
| Gender_Female | .431 | .496 |
| Education* | 2.63 | .676 |
| Nationality_Swedish | .754 | .431 |
|  |  |  |
| *Education: Highest level - | 1 | School |
|  | 2 | College |
|  | 3 | Undergraduate |
|  | 4 | Postgraduate |
|  | 5 | Doctorate |

**Ecological Model**

The model assumes that harvest from resources A and B can, for each resource, be described by the maximum sustainable yield equations (EQ A). The model is the same as in [1]. The maximum harvest of 50 units can only be obtained if resource B is harvested optimally and vice versa. If resource B is not harvested optimally, the harvest of resource A is described by a lower curve. The details of the resource dynamics of A and B are described below.

SY = E*K*(1-E/R) (EQ A)

(SY = Sustainable yield, E=Effort, K=Carrying Capacity, R=Growth rate).

Max sustainable yield (MSY) is when E=R/2 and thus equals R*K/4.

In our model, R is set to 20, which gives best E=R/2 = 10. Furthermore, K is set to 10, which then give that MSY = R*K/4 = 20*10/4 = 50. However, if the other resource is not optimally managed, i.e. E is not equal to R/2 for that resource, the carrying capacity K of resource in question is reduced. K is reduced from its max (10) depending on how far away from R/2 the chosen E is for the other resource. The reduction of K follows a Gaussian curve (EQ. B) with its mean at E=R/2 and with a standard deviation of 10. At E=R/2 (the mean) the Gaussian curve would with this standard deviation be 0.0399, but since we want K at the optimal to be 10, we need to multiply the gauss curve with a normalizing constant so that at E=R/2, the product of the Gaussian curve and the constant would equal maximum K, i.e. 10.

Gaussian curve = exp(-(Et -mean)^2/2/STD^2)/ Sqr(2 * PI * STD ^ 2) (EQ B)

(Et = an instance of chosen E at time t, mean = R/2, STD (standard deviation) = 10)

Since we want to have different optimal levels of E for different players, we added an offset to the chosen Et. These offsets were chosen so that the yellow player’s optimal E would be 11 and the blue/red player’s optimal was 14. In that way the optimal value E varied for different players, although the underlying equations were the same.

For resource A (the resource shared between the blue and the red player), the players’ efforts are added to attain the total E (optimally the combined efforts should be 14). The harvest is shared according to each player’s share of the total E.

**Results from the random effect panel data regression models**

The results are presented for the predictor variables time (round) and total number of messages (per round).

# **Regression with communication and external information**

Oneway (individual) effect Random Effect Model

(Swamy-Arora's transformation)

Call:

plm(formula = optimal_diff ~ totmsg + as.factor(round), data = Info,

na.action = na.omit, model = "random", index = c("agame",

"round"))

Balanced Panel: n=19, T=22, N=418

Effects:

var std.dev share

idiosyncratic 1.987 1.410 0.615

individual 1.244 1.115 0.385

theta: 0.7399

Residuals :

Min. 1st Qu. Median 3rd Qu. Max.

-3.540 -0.889 -0.234 0.589 7.290

Coefficients :

Estimate Std. Error t-value Pr(>|t|)

(Intercept) 3.5387540 0.4357481 8.1211 5.958e-15 ***

totmsg 0.0014735 0.0093042 0.1584 0.8742437

as.factor(round)2 0.3678006 0.4564981 0.8057 0.4209004

as.factor(round)3 0.3494107 0.4565761 0.7653 0.4445590

as.factor(round)4 0.0315995 0.4570128 0.0691 0.9449103

as.factor(round)5 -0.6088207 0.4630741 -1.3147 0.1893611

as.factor(round)6 -0.4137425 0.4610352 -0.8974 0.3700412

as.factor(round)7 -1.0373333 0.4567021 -2.2714 0.0236628 *

as.factor(round)8 -1.0174646 0.4564815 -2.2289 0.0263806 *

as.factor(round)9 -0.8443438 0.4567021 -1.8488 0.0652360 .

as.factor(round)10 -1.0516957 0.4565191 -2.3037 0.0217564 *

as.factor(round)11 -0.9363409 0.4583309 -2.0429 0.0417220 *

as.factor(round)12 -1.4218910 0.4710039 -3.0189 0.0027019 **

as.factor(round)13 -1.1417381 0.4565663 -2.5007 0.0127988 *

as.factor(round)14 -1.5772359 0.4566084 -3.4542 0.0006116 ***

as.factor(round)15 -0.8113483 0.4573042 -1.7742 0.0768006 .

as.factor(round)16 -0.7352082 0.4565971 -1.6102 0.1081551

as.factor(round)17 -1.2097455 0.4565075 -2.6500 0.0083725 **

as.factor(round)18 -1.4369556 0.4565971 -3.1471 0.0017741 **

as.factor(round)19 -1.7375401 0.4565025 -3.8062 0.0001635 ***

as.factor(round)20 -0.9405078 0.4614563 -2.0381 0.0422026 *

as.factor(round)21 -1.4198840 0.4565403 -3.1101 0.0020059 **

as.factor(round)22 -0.9261654 0.4570611 -2.0263 0.0434005 *

---

Signif. codes: 0 ‘***’ 0.001 ‘**’ 0.01 ‘*’ 0.05 ‘.’ 0.1 ‘ ’ 1

Total Sum of Squares: 921.47

Residual Sum of Squares: 781.93

R-Squared: 0.15143

Adj. R-Squared: 0.1431

F-statistic: 3.20415 on 22 and 395 DF, p-value: 2.3451e-06

# **Regression with communication**

Oneway (individual) effect Random Effect Model

(Swamy-Arora's transformation)

Call:

plm(formula = optimal_diff ~ totmsg + as.factor(round), data = noInfo,

na.action = na.omit, model = "random", index = c("agame",

"round"))

Balanced Panel: n=8, T=22, N=176

Effects:

var std.dev share

idiosyncratic 0.8897 0.9432 0.802

individual 0.2202 0.4692 0.198

theta: 0.6061

Residuals :

Min. 1st Qu. Median 3rd Qu. Max.

-2.210 -0.543 -0.162 0.403 4.210

Coefficients :

Estimate Std. Error t-value Pr(>|t|)

(Intercept) 3.5996219 0.4001703 8.9952 8.518e-16 ***

totmsg 0.0021148 0.0129449 0.1634 0.8704408

as.factor(round)2 -0.7157395 0.4691518 -1.5256 0.1291733

as.factor(round)3 -1.3325319 0.4669842 -2.8535 0.0049235 **

as.factor(round)4 -1.1199772 0.4679700 -2.3933 0.0179110 *

as.factor(round)5 -0.8776311 0.4672393 -1.8783 0.0622382 .

as.factor(round)6 -1.5806814 0.4672393 -3.3830 0.0009105 ***

as.factor(round)7 -1.3006506 0.4701887 -2.7662 0.0063699 **

as.factor(round)8 -1.6735357 0.4688504 -3.5694 0.0004783 ***

as.factor(round)9 -1.3765986 0.4670599 -2.9474 0.0037073 **

as.factor(round)10 -1.7157145 0.4691518 -3.6571 0.0003504 ***

as.factor(round)11 -2.0031723 0.4673625 -4.2861 3.204e-05 ***

as.factor(round)12 -1.9219496 0.4680791 -4.1060 6.531e-05 ***

as.factor(round)13 -1.9151014 0.4670599 -4.1003 6.678e-05 ***

as.factor(round)14 -2.0480195 0.4685711 -4.3708 2.275e-05 ***

as.factor(round)15 -1.8046284 0.4736425 -3.8101 0.0002008 ***

as.factor(round)16 -2.2062227 0.4671384 -4.7228 5.220e-06 ***

as.factor(round)17 -2.1743413 0.4693109 -4.6331 7.655e-06 ***

as.factor(round)18 -2.3325444 0.4669842 -4.9949 1.589e-06 ***

as.factor(round)19 -2.3375672 0.4676762 -4.9983 1.565e-06 ***

as.factor(round)20 -2.1276561 0.4672393 -4.5537 1.069e-05 ***

as.factor(round)21 -2.2138765 0.4681937 -4.7285 5.094e-06 ***

as.factor(round)22 -2.2916625 0.4669590 -4.9076 2.338e-06 ***

---

Signif. codes: 0 ‘***’ 0.001 ‘**’ 0.01 ‘*’ 0.05 ‘.’ 0.1 ‘ ’ 1

Total Sum of Squares: 194.87

Residual Sum of Squares: 133.45

R-Squared: 0.31522

Adj. R-Squared: 0.27402

F-statistic: 3.20128 on 22 and 153 DF, p-value: 1.3121e-05

**References**

1. Lindahl T, Bodin Ö, Tengö M. Governing complex commons – the role of communication for experimental learning and coordinated management. Ecol Econ. 2015;111: 111–120. doi:10.1016/j.ecolecon.2015.01.011
